# Inhalation of a Short-Acting β_2_-Adrenoreceptor Agonist Induces a Hypercoagulable State in Healthy Subjects

**DOI:** 10.1371/journal.pone.0158652

**Published:** 2016-07-05

**Authors:** Mais Ali-Saleh, Galit Sarig, Jacob N. Ablin, Benjamin Brenner, Giris Jacob

**Affiliations:** 1 Internal Medicine and J. Recanati Autonomic Dysfunction Center, Tel Aviv "Sourasky" Medical Center, Faculty of Medicine, Tel Aviv University, Tel Aviv, Israel; 2 Thrombosis and Hemostatic Unit, Rambam Medical Center, Ruth and Bruce Rappaport Faculty of Medicine, Technion-IIT, Haifa, Israel; Harvard Medical School, UNITED STATES

## Abstract

**Background:**

Catecholamine infusion elicits an increase in clotting factors and this increase has been attributed to stimulation of β_2_-adrenorecptors (β2AR). Accordingly, we tested the hypothesis that inhalation of a short-acting selective β2AR agonist can induce a procoagulant state in healthy individuals.

**Methods:**

We recruited 23 healthy volunteers (nine females; mean age: 26±0.8 years; body mass index: 24.7±0.5 kg/m^2^) and randomly allocated them into two groups, the β2AR arm (seventeen subjects) and the saline arm (six subjects). Hemodynamics, plasma norepinephrine concentration, and procoagulant, anticoagulant, and fibrinolytic profiles of each participant were determined using specific assays before and after inhalation of either 2 mL nebulized normal saline or a mixture of 1 mL saline and 1 mL of salbutamol (5 mg salbutamol sulfate), a selective β2AR agonist, which were delivered by a nebulizer over ten minutes.

**Results:**

Saline inhalation had no effect on the procoagulant, anticoagulant and fibrinolytic profiles of the six healthy volunteer in the study's saline arm. Salbutamol inhalation caused (a) a significant increase in the activity or levels of the procoagulant factors; FVIII increased by 11±3% (p = 0.04), von Willebrand factor increased by 7±1% (p = 0.03), and (b) a significant decrease in the activated partial prothrombin time from 27.4±0.4 seconds to 25.5 ±0.5 seconds (p<0.001) in the 17 volunteers in the study's β2AR arm. D-dimer and prothrombin fragments F1+2 were elevated by 200 ±90% and 505.0 ±300.0%, respectively. In addition, the activity of the anticoagulant protein C pathway (demonstrated by the protein C Global assay) decreased from 1.0±0.08 to 0.82±0.06 (p<0.001). Although plasma levels of tissue plasminogen activator decreased, all other indices of the fibrinolytic system did not change following salbutamol inhalation.

**Conclusion:**

We found that a single inhalation of salbutamol, a short-acting β2AR agonist, activates the clotting system without affecting the fibrinolytic system. This induction of a procoagulant state in healthy subjects warrants further investigation in patients treated with these agents.

## Introduction

The autonomic nervous system has an essential role in maintenance of cardiovascular homeostasis. Any imbalance in its activity, such as continuous stimulation of the sympathetic nervous system in chronic stress, hypertension, renal failure and structural heart disease [1–5], increases the risk of cardiovascular morbidity and mortality [6]. Increased oxidative stress and dysfunction of the vascular endothelium are also frequently present in conditions of imbalance of the sympathetic nervous system and their presence can manifest as profound disturbances in the coagulation/fibrinolysis balance [7, 8]. Although the association between the disturbed sympathetic nervous system and blood coagulation/fibrinolytic systems are known, its direct effects have not been closely scrutinized. Hence, exploring the direct relationship between adrenoreceptor activation and coagulation and fibrinolytic systems could shed light on some pathophysiologic aspects of this connection.

It is known that the coagulation cascade can be activated by the sympathetic nervous system [8, 9]. Recently, we reported that prolonged orthostatic stress in healthy volunteers can induce a procoagulant state, partially induced by sympathetic activation [10, 11]. Epinephrine (adrenaline) has been reported to initiate coagulation in humans by triggering the release of von Willebrand factor (vWF) and factor VIII and by activating platelets. The increased release of vWF and factor-VIII, the increase in tissue-type plasminogen activator (tPA) and the decrease in plasminogen activator inhibitor (PAI-1) are achieved by β2-adrenoceptors (β2AR) stimulation. These effects can also be obtained following an adrenaline and an isoproterenol infusion, but not following a norepinephrine (NE) infusion because NE is not a β2AR agonist [5, 12, 13]. Anecdotal reports indicates that intravenous salbutamol, a pure β2AR agonist, in humans can increase procoagulant factors, vWF and clotting factor VIII [14]. Furthermore, the results of several in vitro and in vivo studies showed that propranolol, a non-selective βAR antagonist, was able to abolish the adrenaline-induced release of vWF from endothelial cells, an effect which was not achieved by a non-selective αAR antagonist [14, 15]. Propranolol has also been demonstrated to decrease elevated factor VIII in patients with venous thromboembolism [15].

Thus, sympathetic activation causes platelet activation and aggregation by an α2AR-mediated pathway [16] and activates the coagulation/fibrinolytic system through a β2AR-mediated pathway [2, 17, 18]. Since the effect of a β2AR agonist on the coagulation/fibrinolysis balance has been investigated in only a few subjects, using intravenous salbutamol infusion, no data exists regarding the effect of an inhaled β2AR agonist on this balance. Patients with asthma or chronic obstructive pulmonary disease (COPD) are at high risk for pulmonary thromboembolism [19]. Accordingly, we posited that the inhalation of salbutamol, a short-acting selective β2AR agonist, induces a procoagulant state in healthy subjects.

## Methods

The study was approved by the IRB of the Tel Aviv Sourasky medical center and all participants gave written informed consent.

The investigation was a prospective, randomized, single-blind study.

### Subjects

Participants were recruited by advertising. Eligibility criteria included: generally healthy, aged 2–45, and body mass index (BMI) 18–25 kg/m^2^. Exclusion criteria included pregnancy, oral contraceptive within 3 months, history of abortion, recent illness and medication, personal or family history of hypercoagulability or thrombosis, asthma, tachycardia, anxiety disorder or cancer.

Participants were trained to use a hand-held inhaler, connected to a compressed air cylinder. Female participants were tested one week post- menstruation.

### Experimental Design

Measurements were performed between 0800 and 1000 to avoid diurnal fluctuations of the coagulation system after an overnight fast in a quiet, partially darkened and air-conditioned room with an ambient temperature of ~24°C. On the day of testing, each subject was placed in a supine position with the head elevated at 30 degrees. A 21G heparin-free lock was inserted into a large antecubital vein and flushed with 3 mL of heparin-free normal saline. Heart rate (HR) and blood pressure (BP) were monitored continuously. Upon completion of a 30-minute rest period, a 20-mL blood sample (5 mL for determining plasma NE concentrations and 15 mL for determining the procoagulant, anticoagulant and fibrinolytic profiles) was collected from each subject after discarding the first 2 mL of blood. After a 15-minute rest period, subjects inhaled either 2 mL nebulized normal saline or a mixture of 1 mL saline and 1 mL of salbutamol (5 mg salbutamol sulfate) delivered by a nebulizer over ten minutes. After resting for 45 minutes (overall 60 minutes from inhalation beginning), repeat samples were collected, without using a tourniquet and 3 mL heparin-free normal saline was injected after each blood sampling. Since the time to reach peak plasma levels of inhaled salbutamol is about ~15 minutes, we chose to collect the blood samples at least 45 minutes after the end of the salbutamol inhalation (~ 60 minutes after collecting the baseline sample).

### Determination of the Procoagulant, Anticoagulant and Fibrinolytic Profiles

The procoagulant, anticoagulant and fibrinolytic profiles were determined in each participant. The procoagulant pathway included prothrombin time (PT), activated partial PT time (aPTT), fibrinogen levels, factor V and factor VIII activities, and von Willebrand factor (vWF) antigen. The activity of the protein C anticoagulant pathway was evaluated using the ProC Global assay. Other specific markers of coagulation activation and thrombin activation, such as prothrombin fragments F1+2 and D-dimer and fibrin formation and fibrinolysis, were also measured. Analysis of the fibrinolytic system included plasma concentrations of tissue plasminogen activator (tPA) and its inhibitor, plasminogen activator inhibitor (PAI-1). The plasma activities of plasminogen and α2-antiplasmin were also determined following the salbutamol inhalation.

Blood samples were collected into 3.2% sodium citrate tubes and centrifuged at 2000g for 15 minutes. PT, aPTT, fibrinogen, D-dimer and ProC Global assays were performed on fresh plasma samples, whereas all other coagulation assays were done using thawed frozen plasma samples. Plasma samples were frozen at -70±5°C after a second centrifugation at 2000g for 15 minutes in aliquots. Before testing, plasma aliquots were thawed in a warmed (37.0±0.5°C) water bath for 15 minutes. PT, aPTT, and ProC Global assays were performed on the Sysmex CA-7000 system (Siemens Healthcare Diagnostics, Marburg, Germany) using Dade Innovin, Dade Actin FS, Test Thrombin Reagent and ProC^®^ Global (Siemens Healthcare Diagnostics, Marburg, Germany) and STA-LIATEST D-DI kits (Diagnostica Stago) for the D-dimer assays. The ProC Global assay (Dade Behring) was performed as previously described [20, 21]. Levels of vWF antigen, plasminogen and α2-antiplasmin and coagulation factors V and VIII activity were determined on the STA-R Evolution analyzer (Diagnostica Stago, Asnières, France) using STA-Lia test vWF antigen, STA-Stachrom plasminogen and antiplasmin kits, respectively (Diagnostica Stago, Asnières, France). The activity of factors V and VIII were determined by a one-stage assay using factor V and factor VIII respectively deficient plasma (Diagnostica Stago, Asnières, France).

The prothrombin fragment F1+2 concentration was measured by an enzyme immunoassay (ELISA) using Enzygnost* F1+2 (Siemens Healthcare Diagnostics, Marburg, Germany). The plasma antigen concentrations of PAI-1 and t-PA were determined by ELISA using Asserachrom^®^ PAI-1 and tPA kits respectively (Diagnostica Stago, Asnières, France).

### Determination of Plasma NE Concentrations

A 5-mL blood sample was collected in a plastic syringe and then immediately transferred to a chilled EDTA vacuum tube which was immediately placed in crushed ice. Blood was separated by refrigerated centrifugation at -4°C and the plasma stored at -70°C. Plasma NE concentration was measured as previously described [22].

### Statistical Analysis

Data were analyzed using Excel (Microsoft, Redmond, WA, USA) and GraphPad Prism version 6.02 for Windows (GraphPad Software Inc., La Jolla, CA, USA). Results are presented as mean ± standard error of the mean and statistical significance was set at 5%. Parametric data were analyzed using a paired two tailed t-test for intra- and intergroup comparisons. Non-parametric data were analyzed using the paired Mann-Whitney U test for comparisons between changes of the coagulation factors. The sample size was powered for the β2AR group (80%, α<0.05), but not for the saline group.

## Results

We recruited 23 healthy volunteers (nine females), whose general characteristics are displayed in Table 1. The 23 volunteers were randomly allocated into two groups. Group 1 subjects (n = 17) were assigned to the β2AR arm of the study and group 2 subjects (n = 6) were assigned to the saline arm of the study. We stopped the saline arm of the study after testing six subjects because the results of an interim analysis revealed a definitive lack of effect on their procoagulant, anticoagulant and fibrinolytic profiles. Two participants in the β2AR arm reported transient palpitations during the salbutamol inhalation and none of the other participants reported any adverse effects during testing.

**Table 1:** The general characteristics of the 23 study participants.

	β2AR group n = 17	Saline group n = 6
*Age*, *years*	26±0.8	27±1
*Female/Male*	7/10	2/4
*Body Mass Index*, *kg/m*^*2*^	23.9±0.6	23.5±0.8
*Baseline Systolic Blood Pressure*, *mm Hg*	116±3	115±3
*After a 45-minute rest*, *mm Hg*	*120±3*	*113±4*
*Baseline Diastolic Blood Pressure*, *mm Hg*	69±3	71±3
*After a 45-minute rest*, *mm Hg*	*66±2*	*72±3*
*Baseline Heart Rate*, *beats/minute*	68±3	66±2
*After a 45-minute rest*, *beats/minute*	*76±2*	*65±4*

**Fig 1:**
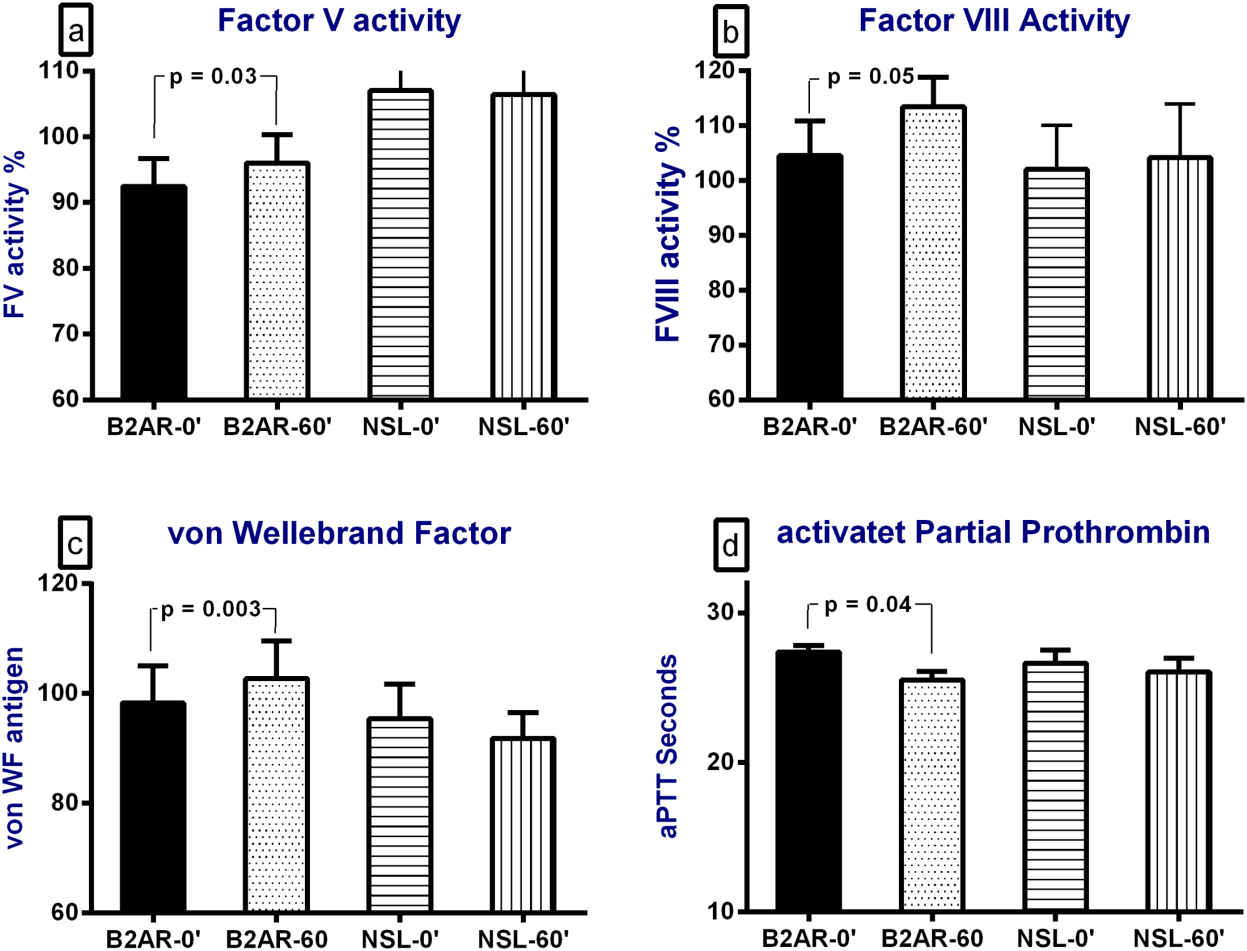
The effect of a single inhalation of the selective short-acting β2-adrenorecptor (B2AR) agonist, salbutamol, and normal saline (NSL) on (a) plasma factor V activity levels, (b) plasma factor VIII activity levels, (c) plasma von Willebrand antigen levels and (d) the activated partial prothrombin time (aPTT). "0" for baseline and "60" for one hour after the start of the inhalation.

**Fig 2:**
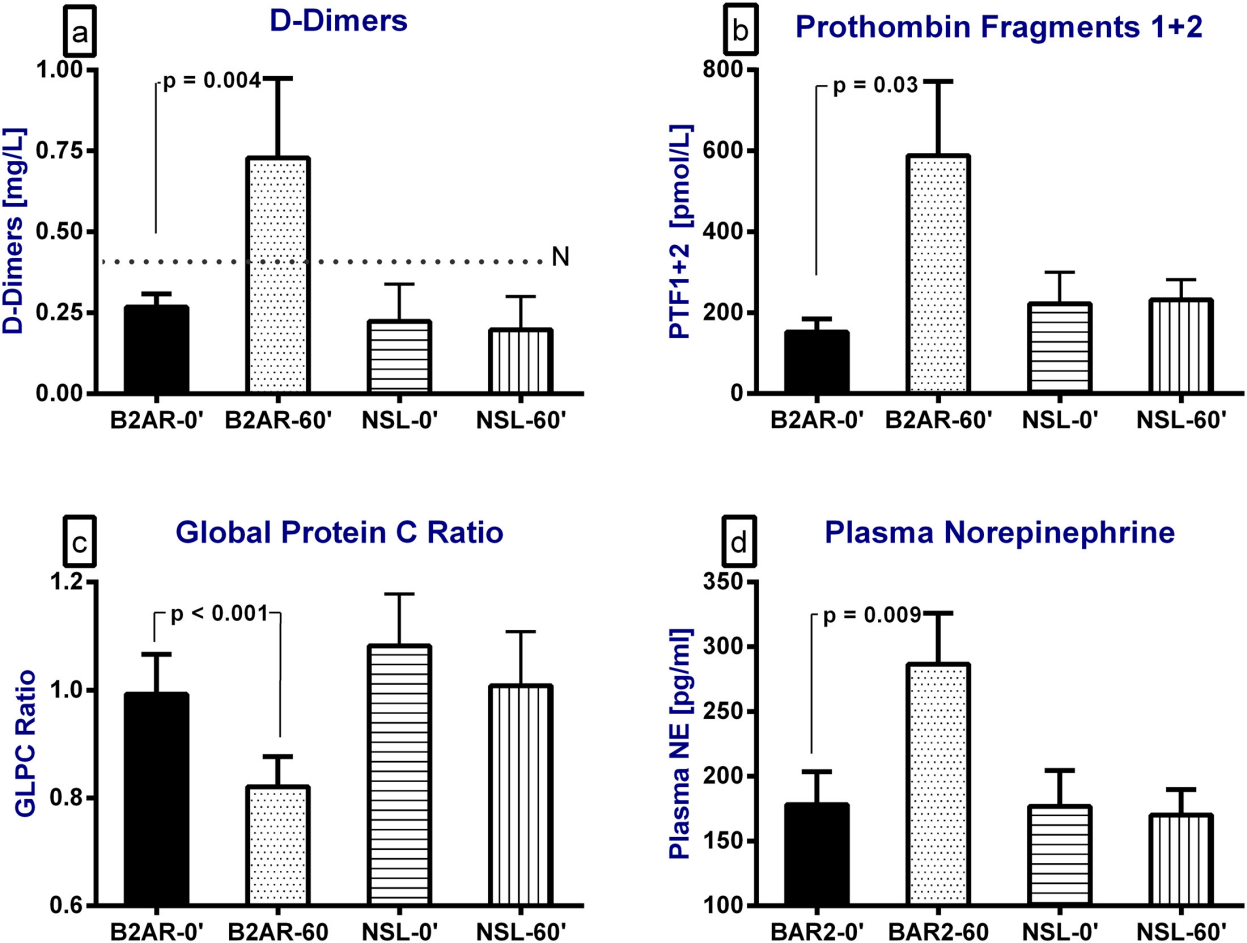
The effect of a single inhalation of the selective short-acting β2-adrenorecptor agonist (B2AR), salbutamol, and normal saline (NSL) on (a) plasma D-dimer levels, (b) prothrombin degradation fragments 1+2, (c) the Global Protein C (GLPC) ratio, a measure of the activated Protein C complex activity and (d) the plasma norepinephrine (NE) concentration. "0" for baseline and "60" for one hour after the start of the inhalation.

Salbutamol inhalation caused significant increases in the plasma levels of the procoagulant markers, namely plasma FV activity (p = 0.03), FVIII activity (104±6% to 114±5%, p = 0.05) and plasma vWF antigen levels (p = 0.003) (Fig 1a–1c) and the plasma levels of fibrin degradation markers, namely plasma D-dimer levels (0.26±0.04 to 0.73±0.25 mg/L, p<0.001) and plasma prothrombin fragment F1+2 levels (166±32 to 588±180 pmol/L, p = 0.03) (Fig 2a and 2b). These increases were associated with significant decreases in the aPTT (p<0.001; Fig 1d) and protein C global ratio, a marker of the protein C pathway activity (p<0.001; Fig 2c). Salbutamol inhalation caused a significant increase in plasma norepinephrine concentration, from 173±25 to 286±39 pg/ml, p = 0.009, (Fig 2d).

**Fig 3:**
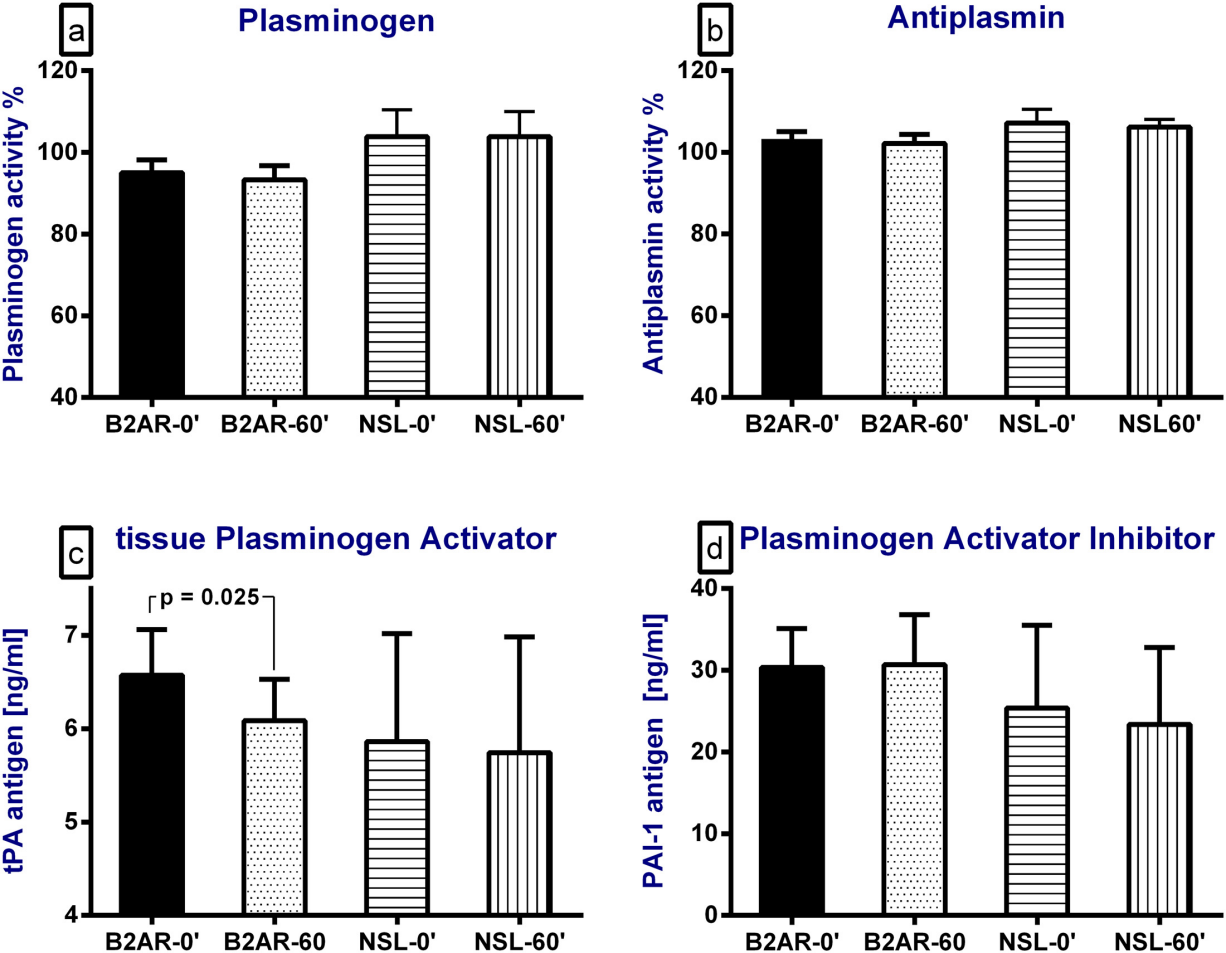
The effect of a single inhalation of the selective short-acting β2-adrenorecptor agonist (B2AR), salbutamol, and normal saline (NSL) on (a) plasma plasminogen activity of, (b) plasma anti-plasmin activity, (c) plasma tissue plasminogen activator (tPA) levels, and (d) plasma plasminogen activator inhibitor-1 (PAI-1) levels. "0" for baseline and "60" for one hour after the start of the inhalation.

No significant changes were detected in plasma levels of the fibrinolysis markers (Fig 3) except for the tPA activity (6.5±0.5 to 0.60±0.4 ng/ml; p<0.025; Fig 3c). Additionally, the tPA/PAI ratio did not change after the salbutamol inhalation (0.32±0.04 vs. 0.34±0.06; p = 0.4). S1 Table.

## Discussion

Our hypothesis that the β2AR is the key regulator of sympathetically-mediated blood coagulation is based on a physiologic and pharmacologic analysis of the reported effects of adrenergic agonists and antagonists on the coagulation system [9]. In this report, we tested this hypothesis in a group of healthy volunteers and found that inhalation of salbutamol, a short-acting selective β2AR agonist, activates the clotting system and reduces the anticoagulant activity of the protein C pathway, without any major effects on the fibrinolytic system, except for tPA.

We found that plasma NE concentrations increase following salbutamol inhalation. A similar effect is reported on epinephrine [23]. This increase in NE is released from the sympathetic nerve endings by activation of presynaptic β2ARs [24, 25]. In addition, baro-reflex-mediated activation of sympathetic nerve activity due to β2AR-mediated peripheral vasodilation also contributes the increased plasma NE concentration.

Consequently, this significant increase in plasma NE concentration, could account for platelet activation in our participants through α2AR, which was not assessed in our study [26].

We also found that vWF levels increase following salbutamol inhalation. Vascular endothelial cells contain Weibel-Palade bodies, which are the storage organelles for vWF, whose function is to control platelet adhesion and aggregation at sites of vascular injury ("biologic glue"). Adrenaline and isoproterenol (through β2AR) and thrombin are known to activate endothelial cells. This activation causes fusion of the Weibel-Palade bodies with the plasma membrane and release of their contents into the blood circulation as part of the response to arrest bleeding following a vascular injury [5, 27]. Accordingly, we surmise that α2AR-mediated platelet activation and β2AR-mediated vWF release could account for activation of the coagulation cascade following salbutamol inhalation.

We found that FVIII and FV levels increased following the salbutamol inhalation. This finding is not surprising because it has been reported that vWF has a protective and stabilizing effect on FVIII [28]. A dose-dependent increase in FVIII levels, which is mediated by β2AR, is a frequent finding in studies which investigated the effects of catecholamines on coagulation [12, 14]. A β2AR-mediated increase in FV levels has also been found in such studies [29, 30]. In addition, there are anecdotal reports that the increase in FVIII levels following salbutamol intravenous infusion occurs without any effect on other coagulation factors [12, 14].

We also found that this β2AR-induced increase in procoagulant factors following salbutamol inhalation is also associated with a decrease in the aPTT. The effects of epinephrine on aPTT are contradictory. Two studies showed an increase and one reported no change in aPTT following an epinephrine infusion [31–33].

Prothrombin activation fragments, such as F1+2, a marker of thrombin generation, and fibrinogen degradation products, such as D-dimers are very specific coagulation activation markers. The most conspicuous piece of evidence for a β2AR-induced procoagulant state following the salbutamol inhalation in this study is the resultant increase in these coagulation activation markers. Although this is a novel finding, it has been reported that an epinephrine infusion increases the total thrombin—anti-thrombin complex (TAT) without affecting plasma F1+2 levels [34]. It has also been reported that there is a positive correlation between increases in F1+2 levels and a metabolite of NE [35]. This increase in F1+2 levels was interpreted as being due to α2AR-mediated platelet activation. On the other hand, von Känel et al. have previously reported that isoprenalin, a nonselective βAR agonist, decreases D-dimer concentration in hypertensive subjects [5].

We found reduced activity of the global protein C pathway, as measured by a low GLPC ratio, following salbutamol inhalation. Since we found that salbutamol inhalation induces a procoagulant state, we surmise that the reduced activity of this endothelial-dependent anticoagulant system could be due to over consumption because of the significant increases in FV and FVIII activities following salbutamol inhalation. Activated FV and FVIII are the main factors which accelerate thrombin production [10] and the protein C complex, whose essential components are the endothelial cell protein C receptor, proteins C and S, thrombin and thrombomodulin, is their physiological antagonist [36]. The degree of activation of the coagulation system directly influences the degree of utilization of protein C complex and this relationship is expressed in the GLPC ratio: a low ratio implies intense activation of the coagulation system and increased utilization of the protein C complex.

The connection between the protein C complex and the autonomic nervous system has not been extensively investigated. It has been reported that inhalation of a long-acting β2AR agonist by healthy volunteers resulted in reduced protein C activity in broncho-alveolar lavage fluid, with no report on plasma levels [37]. NE and epinephrine infusion reduced plasma protein C levels in sepsis patients [38] and NE can downregulate protein S expression in cultured endothelial cells, an effect which is mediated by α1AR, but not by β2AR or α2AR [39]. No data on the effect of α1AR on the coagulation system in humans exists. Therefore, it remains to be determined whether the decrease in GLPC ratio following salbutamol inhalation is due to increased protein C complex utilization or/and adrenoceptor-mediated suppression of activation of the coagulation systems.

We found that a salbutamol inhalation exerts no major effects on the complex fibrinolytic system, whose activity can be modulated by the autonomic nervous system. The sympathetic effects on the fibrinolytic system are mediated directly by stimulating β2AR on endothelial cells and indirectly by α2AR-mediated platelet activation. It has been reported that an epinephrine or an isoproterenol infusion, but not an NE infusion, increases plasma tPA levels and activity in a dose-dependent manner and decreases plasma PAI-1 activity without changing its plasma concentration [2, 40, 41]. Since we found that a salbutamol inhalation exerts no major effects on the fibrinolytic system, we surmise that a single dose of an inhaled β2AR agonist is not sufficient to effect the fibrinolytic system. A possible explanation for this lack of effect of salbutamol infusion on the fibrinolytic system could be due to the short half-life of tPA (four minutes) and PAI-1 (seven minutes)[42]: we collected blood samples 45 minutes after the end of salbutamol inhalation from the study's participants and the time of this collection is long after tPA and PAI-1 have been cleared from the plasma and any effects would not be detected. Why then were we able to detect an increase in vWF levels following the salbutamol inhalation? The half-life of vWF is about 12 hours and therefore is not cleared from the plasma, 45 minutes after the end of the salbutamol inhalation. Future study using long-acting β2AR agonists (LABA) could shed light on this issue.

Chronic obstructive pulmonary disease (COPD) has been recently defined as an independent risk factor for pulmonary embolism and patients with COPD are frequently prescribed β2AR agonists as a bronchodilator. Our results should not be over-interpreted as representing the sole mechanism responsible for this thromboembolic diathesis but further investigation is warranted [43].

The FDA has recommended that patients with asthma simultaneously use corticosteroids when inhaling long-acting β2AR agonists [44]. There are many reports which provide definitive evidence on the importance of the coagulation system in the pathogenesis of asthma. Some products of the coagulation cascade are involved in triggering the allergic-inflammatory process in asthma [45], and glucocorticosteroids are known to protect against coagulation-induced inflammation in asthma [46, 47]. Notably glucocorticosteroids also have a significant pro-coagulant activity [48]. Hence, we must further understand the balance between the procoagulant and anticoagulant systems in asthma patients.

### Limitations

Although discontinuation of the saline arm (not powered) may have weakened the conclusion, the absence of any effect was evident. We studied the coagulation system by collecting blood samples from participants 60 minutes after the start of the salbutamol inhalation. By doing so, information on the dynamic interaction between β2AR and coagulation could have been masked because of the different half-lives of the coagulation system markers studied. The solution of salbutamol sulfate contains a preservative, 0.01% benzalkonium chloride. Although there is no published information on the effects of benzalkonium chloride on blood coagulation, we cannot exclude such an effect.

In conclusion, we inform on the results of an investigation in which we tested the hypothesis that inhalation of salbutamol, a short-acting selective β2AR agonist, induces a procoagulant state in healthy subjects. We found that a single salbutamol inhalation induces a procoagulant state in healthy volunteers. The clinical significance of this finding should be explored.

## Supporting Information

S1 Table Coagulation data. (XLSX)
